# Age-Related Changes in Human Schlemm’s Canal: An *in Vivo* Optical Coherence Tomography-Based Study

**DOI:** 10.3389/fphys.2018.00630

**Published:** 2018-06-05

**Authors:** Yujin Zhao, Junyi Chen, Xiaobo Yu, Jianjiang Xu, Xinghuai Sun, Jiaxu Hong

**Affiliations:** ^1^Department of Ophthalmology and Visual Science, Shanghai Eye and ENT Hospital, Shanghai Medical College, Fudan University, Shanghai, China; ^2^Department of Ophthalmology, The Affiliated Hospital of Guizhou Medical University, Guiyang, China; ^3^Key Laboratory of Myopia, National Health Commission, Beijing, China; ^4^Leiden Academic Centre for Drug Research, Leiden University, Leiden, Netherlands

**Keywords:** Schlemm’s canal, healthy human, anatomy, spectral-domain optical coherence tomography, agerelated changes

## Abstract

**Purpose:** To investigate age-related changes in human Schlemm’s canal (SC) using spectral-domain optical coherence tomography (SD-OCT).

**Methods:** A total of 125 normal eyes were imaged using SD-OCT nasally and temporally. The age-related variations of SC sagittal diameter and cross-sectional area (CSA) from four age groups [A (16–20 years), B (21–40 years), C (41–60 years), and D (61–80 years)] were analyzed with Spearman correlation.

**Results:** The positive detection rates of SC showed a significantly downward trend with age. The mean CSA was 13,296 ± 1,897 μm^2^ nasally and 14,552 ± 2,589 μm^2^ temporally. The mean CSA was significantly larger in the temporal than in the nasal region (*P* < 0.05). Nasal CSA values varied among the four age groups (*P* = 0.004).

**Conclusion:** Our study found for the first time that SC *in vivo* exhibits a morphological variant with age in healthy humans. Clinicians may need to consider this phenomenon when performing examinations targeting SC for glaucoma patients.

## Introduction

Schlemm’s canal (SC), which runs parallel to the limbus as a circumferential channel, delivers aqueous humor from the anterior chamber into the bloodstream through the anterior ciliary veins. Covered by the trabecular meshwork (TM), SC makes the greatest contribution to the outflow resistance of the aqueous humor. Researchers have determined that most resistance in the aqueous outflow network occurs in the inner wall of the SC (Forgáčová et al., 2013; Yang et al., 2014).

As a non-invasive imaging system for biological tissue scanning, spectral-domain optical coherence tomography (SD-OCT) is increasingly being used to assess the anterior chamber angle in both normal and pathological conditions (Gupta et al., 2017). The ability of SD-OCT to track from SC to superficial vascular plexuses in two-dimensional (2D) image sequences was successfully proven (Kagemann et al., 2012). This system is reliable for *in vivo* SC imaging for both static and dynamic processes (Irshad et al., 2010; Hong et al., 2013, 2016; Xin et al., 2016).

Most of the previous reports that assessed the anatomic characteristics of SC showed age-related variations in SC dimension using various measuring methods. In two reports of postmortem eyes under light and electron microscope observation, the anteroposterior diameter of SC decreased with age, and narrowed canals appeared more frequently in older eyes (Irshad et al., 2010). Ainsworth and Lee (1990) demonstrated an age-related SC size reduction in 20 human donor eyes. However, Irshad et al. (2010) measured the maximum linear SC dimension using ultrasound (80-MHz and ultrasound probe) and found no connection between SC diameter and age. None of the studies noted above involved age-related changes in the cross-sectional area (CSA). The dimension of SC varies under different circumstances. In healthy subjects who suffered from acute intraocular pressure (IOP) elevation, as well as glaucoma eyes, decreased SC dimensions were reported (Forgáčová et al., 2013; Hong et al., 2013; Kagemann et al., 2014b; Xin et al., 2016). According to a recent research, only the CSA and the diameter of SC at the inferior quadrant were negatively associated with IOP changes (Gao et al., 2017). Moreover, mutations of some genes, such as *CYP1B1* and *MYOC*, were reported to be associated with the morphology of SC in congenital and juvenile glaucoma cases (Choudhary et al., 2009; Hamanaka et al., 2017).

Accompanied by unneutralized or accumulated reactive oxygen species, which would cause molecular damage, aging could lead to structural and functional changes in a series of ocular structures (reviewed in Girkin et al., 2011; Babizhayev, 2012; Day et al., 2013; Lei et al., 2013; Zhao et al., 2014). The influence of oxidative stress associated with increasing oxidant production in cells characterized by the release of free radicals, leading to cellular degeneration, is involved in many age-dependent ophthalmic diseases, such as senile cataract and primary open-angle glaucoma (POAG). Oxidative stress in glaucoma pathogenesis and age-related changes of the TM were first proposed in 1981 (Pang et al., 2015). According to previous reports, the onset and progression of glaucoma were accompanied by the accumulation of oxidative damage to the targeted TM (Babizhayev, 2012; Lei et al., 2013; Yang et al., 2014). Trabecular or SC endothelial cell dysfunction and the elevation of outflow resistance have been reported to be related to aging. Other age-related changes in aqueous humor dynamics have been proposed, such as the decline of aqueous production rate, episclera venous pressure, ocular rigidity, and accumulation of extracellular material in the conventional and unconventional outflow pathways (Lei et al., 2013). Boldea et al. (2001) proposed an age-related reduction in counts of giant vacuoles and intracellular pores which reduced the endothelial inner wall outflow ability and influenced SC filtration function. Although some studies have proposed that SC size decreased with age (Irshad et al., 2010; Lei et al., 2013), this has not yet been sufficiently proven. The aim of the current study is to explore the age-related morphological changes of *in vivo* SC in a healthy population using SD-OCT.

## Materials and Methods

### Patients

A total of 155 eyes from 155 healthy volunteers (72 males, 83 females) from 16 to 79 years of age were enrolled in the study. Considering the impact of ametropia on SC parameters, we exclusively enrolled normal subjects without any history of ocular surgeries or diseases whose myopic refractive error ≤ 2 diopters. Subjects with systemic or ocular conditions which could affect the aqueous outflow pathway microstructures, such as glaucoma, intraocular neovascular diseases, and anterior segment congenital anomalies, were excluded. Based on the examples of similar studies (Zheng and Xu, 2008; Zhu et al., 2010), data were collected only from the right eye of each subject. Subjects were divided into four groups according to their age: Group A (16–20 years; 19 males, 13 females; average 18.2 ± 1.3 years), Group B (21–40 years; 19 males, 21 females; average 29.8 ± 7.1 years), Group C (41–60 years; 19 males, 24 females; average 49.4 ± 4.8 years), and Group D (61–80 years; 15 males, 25 females; average 71.0 ± 5.7 years). Informed consent was obtained from all participants and/or their legal guardians after receiving detailed explanations about the nature of this study. The investigation protocols were approved by the Ethics Committee of the Shanghai Eye, Ear, Nose, and Throat Hospital of Fudan University, China. Our study adhered to the tenets of the Declaration of Helsinki. Moreover, all the clinical operations were carried out strictly in accordance with relevant guidelines and regulations.

For all participants, a series of basic comprehensive ophthalmologic examinations, including visual acuity (VA), slit-lamp biomicroscopic examination, IOP measurement, fundus examination, refractive status, and axial length (AL), were performed by one masked practical ophthalmologist (JX). Furthermore, central corneal thickness (CCT), nasal and temporal peripheral corneal thickness (PCTn, and PCTt) were measured and recorded. In detail, Snellen letter charts, an autorefractometer (KR-8100; Topcon, Tokyo, Japan), and a retinal camera (TRC-NW200; Topcon) were used for measurement of VA, refractive status, and fundus state, respectively. An A-scan ultrasound (A-Scan Pachymeter Ultrasonic; DGH Technology, Exton, PA, United States) was applied to measure AL and CCT values. IOP was measured by a Goldmann applanation tonometer (Perkins Tonometer MK2, Haag Streit, United States).

### Instrumentation and Procedures

**FIGURE 1:**
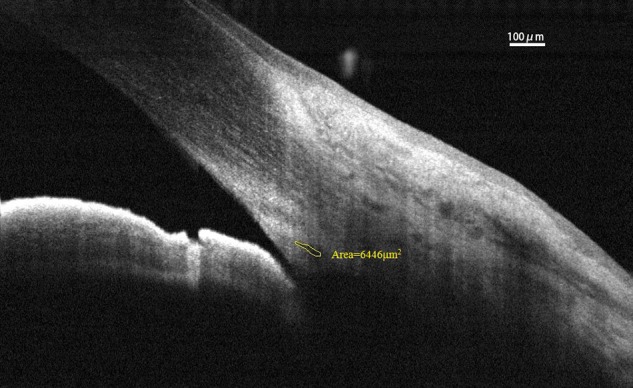
Schlemm’s canal sagittal diameter was obtained as the average value from three independent measurements. SC, Schlemm’s canal.

**FIGURE 2:**
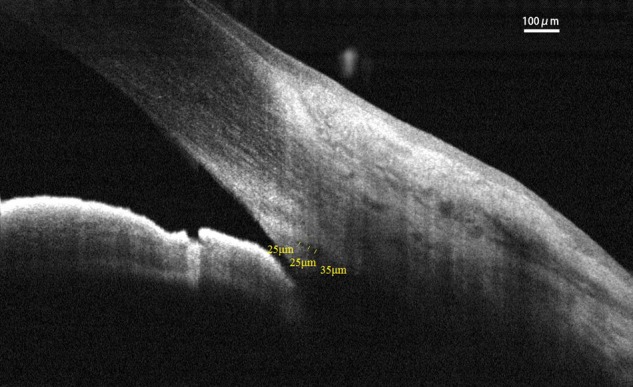
Cross-sectional area (CSA) was defined as the region inside of the outline of the SC. CSA, cross-sectional area; SC, Schlemm’s canal.

Spectral-domain optical coherence tomography (RTvue OCT; Optovue, Toledo, OH, United States) uses the standard anterior-segment single-scan protocol. This instrument collects over 25,000 A-scans per sec on a 6-mm line at an axial resolution of 5 μm. The bright indoor light conditions were kept standardized and consistent throughout the entire process, since pupil dilation can alter the structure of the angle. Every line-scan image was achieved by automatically averaging 16 images into 1 final version to lessen speckle noise after a full blink (as specified in the RTVue user’s manual provided by Optovue Inc., Fremont, CA, United States). For consistency, SC parameters were measured by a single experienced ophthalmologist (YZ). The measuring methods used for the SC sagittal diameter and CSA were completely consistent with those described in our previous studies (Hong et al., 2013; Zhao et al., 2014). The nasal SC sagittal diameter (SCn diameter), temporal SC sagittal diameter (SCt diameter), nasal CSA (CSAn), and temporal CSA (CSAt) were quantified by the software within the device and manually determined by two independent observers (YZ, JC) who were blind to the identities of the subjects. The images were mathematically corrected in accordance with the curvature of the anterior corneal surface as well. Specifically, each SC sagittal diameter was obtained as the average value from three independent measurements of the same SC (**Figure 1**). The CSA was defined as the long circular region inside of the SC outline, and the mean value of CSA was obtained from three repeated measurements (**Figure 2**).

Acceptable repeatability and reproducibility of the current study using this SD-OCT device were proven in our previous reports (Hong et al., 2013, 2016).

### Statistical Analysis

Among 155 healthy subjects who were initially enrolled, 125 right eyes with observable SCs were finally admitted to this study. All the data were submitted to statistical analysis related to age using IBM SPSS Statistics ver. 18.0 (IBM, Armonk, NY, United States). Basic descriptive statistics were conducted on all of the collected information. The chi-square test was applied to analyze the positive detection rate of SC in different age groups. The Student’s *t*-test or Wilcoxon rank-sum (Mann–Whitney *U*) test was used to analyze the differences between males and females, depending on the normality of the test results. One-way analysis of variance (ANOVA) was used to compare the main SC outcomes among the four age groups. Due to the lack of bivariate normal distribution, the age-related variations of the SC parameters were analyzed using the Spearman correlation (Hong et al., 2013). Results were shown as mean ± standard deviation (SD). The repeatability and reproducibility of measurements were assessed by the coefficient of variation (CV) and the intraclass correlation coefficient (ICC). The CV is defined as the ratio of the SD of measurements to the mean, expressed as a percentage. The ICC measures the proportion of total variability in measurements contributed by variability in measurements among different subjects and was determined using the random-effects mixed model. All *P*-values were considered statistically significant when less than 0.05.

## Results

Schlemm’s canal of 30 subjects could not be identified because of poor OCT image quality. Therefore, only 125 eyes from 125 healthy subjects ranging from 16 to 79 years of age were selected for the current study (average 41.2 ± 20.1 years). To exclude the influence of sex, we compared every parameter between the male and female cohorts, but did not find any significant differences (see **Table 1**).

**Table 1:** Clinical parameters in male and female subjects.

Gender	SCn diameter (μm)	SCt diameter (μm)	CSAn (μm^2^)	CSAt (μm^2^)	CCT (μm)	AL (mm)	IOP (mmHg)	PCTn (μm)	PCTt (μm)
Male	47.7 ± 3.4	48.6 ± 3.4	13,263 ± 1,737	14,737 ± 2,334	554.6 ± 38.5	24.0 ± 2.7	17.1 ± 2.7	862.6 ± 42.0	880.8 ± 41.0
Female	46.3 ± 12.7	47.2 ± 14.0	13,324 ± 2,033	14,397 ± 2,792	552.1 ± 40.4	23.5 ± 1.5	16.0 ± 2.5	855.8 ± 39.0	874.7 ± 43.2
Total	46.9 ± 9.6	47.8 ± 10.6	13,296 ± 1,897	14,552 ± 2,589	554.9 ± 38.7	23.73 ± 2.12	16.5 ± 2.6	858.9 ± 40.3	877.5 ± 42.1
*P*-value	0.228	0.594	0.653	0.487	0.929	0.618	0.115	0.823	0.232

*The Student t-test and the Wilcoxon rank-sum (Mann–Whitney U) test were used. SCn, nasal Schlemm’s canal; SCt, temporal Schlemm’s canal; CSAn, nasal cross-sectional area; CSAt, temporal cross-sectional area; CCT, central corneal thickness; AL, axial length; IOP, intraocular pressure; PCTn, nasal peripheral corneal thickness; PCTt, temporal peripheral corneal thickness*.

In the current study, SC was successfully viewed and analyzed in 30 eyes (93.8%) in Group A, 34 eyes (85.0%) in Group B, 34 eyes (79.1%) in Group C, and 27 eyes (67.5%) in Group D. The positive rates of SC showed an explicitly downward trend with age (see **Table 2**).

**Table 2:** The positive rates of SC in different age groups.

Age Group	Presence	Absence	Total	Positive rate (%)
A	30	2	32	93.8%
B	34	6	40	85.0%
C	34	9	43	79.1%
D	27	13	40	67.5%
	
χ^2^ value	8.503		
*P*-value	0.037		

*The Chi-square test was used. SC, Schlemm’s canal.*

For intraobserver repeatability, the CV and ICC values were 7.7% and 0.96 for the SC diameter, respectively. As for the CSA, the CV and ICC values were 12.3% and 0.86, respectively. For interobserver repeatability, the CV and ICC values were 13.5% and 0.86 for the SC diameter, and 13.1% and 0.82 for the CSA.

The mean diameters of the nasal and temporal sections were 46.9 ± 9.6 μm and 47.8 ± 10.6 μm, respectively. The CSA, depicted as the dark irregular circular space inside of the SC outline, was significantly larger in the temporal area (14,552 ± 2,589 μm^2^ vs. 13,296 ± 1,897 μm^2^), as shown in **Table 3**. Neither CCT nor AL correlated with SC outcomes. Results are shown in **Table 4**.

**Table 3:** Comparison of nasal and temporal Schlemm’s canal morphology.

	SCn diameter (μm)	SCt diameter (μm)	CSAn (μm^2^)	CSAt (μm^2^)
Mean value	46.9 ± 9.6	47.8 ± 10.6	13,296 ± 1,897	14,552 ± 2,589
		
*Z*-value	-0.715		-3.685	
*P*-value	0.476		0.000	

*The Wilcoxon rank-sum (Mann–Whitney U) test was used. SCn, nasal Schlemm’s canal; SCt, temporal Schlemm’s canal; CSAn, nasal cross-sectional area; CSAt, temporal cross-sectional area.*

**Table 4:** Correlation analysis of CCT and AL with Schlemm’s canal parameters.

	SCn diameter	SCt diameter	CSAn	CSAt	PCTn	PCTt
**CCT *r*_s_ (*P*)**	-0.061 (0.498)	0.002 (0.981)	0.070 (0.440)	0.087 (0.334)	-0.099 (0.270)	0.120 (0.181)
**AL *r*_s_ (*P*)**	0.098 (0.278)	-0.014 (0.876)	-0.154 (0.087)	-0.009 (0.919)	0.120 (0.181)	-0.001 (0.994)

*Pearson or Spearman correlation was used. SCn, nasal Schlemm’s canal; SCt, temporal Schlemm’s canal; CSAn, nasal cross-sectional area; CSAt, temporal cross-sectional area; CCT, central corneal thickness; AL, axial length; IOP, intraocular pressure; PCTn, nasal peripheral corneal thickness; PCTt, temporal peripheral corneal thickness.*

**FIGURE 3:**
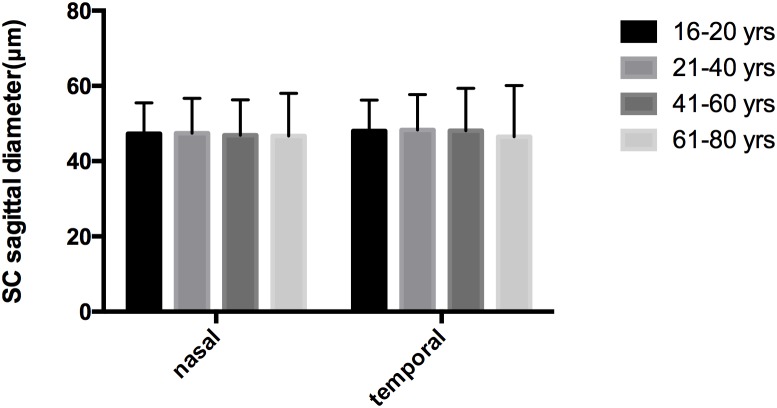
Histogram of SC sagittal diameter among the four age groups. SC, Schlemm’s canal.

**FIGURE 4:**
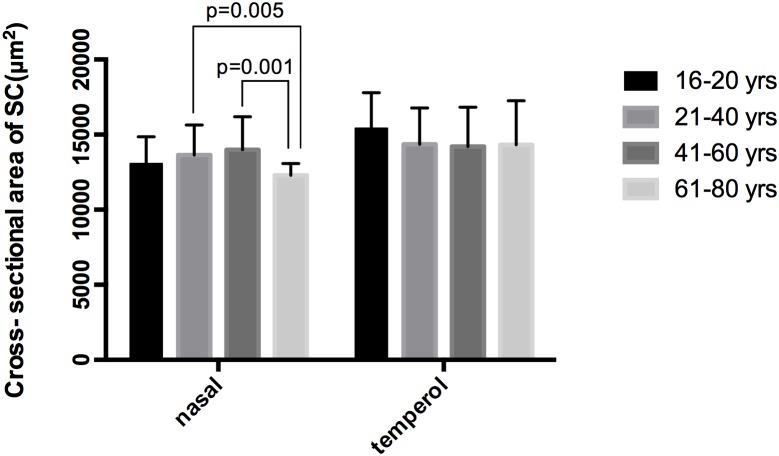
Histogram of CSA among the four age groups. CSA, cross-sectional area.

No significant correlations were observed between aging and SCn diameter (*r* = -0.084, *P* = 0.349), SCt diameter (*r* = -0.037, *P* = 0.683), CSAn (*r* = -0.128, *P* = 0.154) or CSAt (*r* = -0.156, *P* = 0.082) (see **Table 5**, Line 6). However, within the four age groups, we did detect some differences (*F* = 4.658, *P* = 0.004). Specifically, the CSAn value of group D was significantly smaller than that in Groups B (*P* = 0.005) and C (*P* = 0.001). The CSAn value in Group A was not significantly different from those in the other three groups [*P* = 0.785 (Group B), 0.411 (Group C), and 0.212 (Group D)]. No significant difference was found in the CSAn values between Groups B and C (*P* = 0.993) either (see **Figures 3**, **4**).

**Table 5:** Comparisons of Schlemm’s canal outcomes at various ages.

Age group (years)	SCn diameter (μm)	SCt diameter (μm)	CSAn (μm^2^)	CSAt (μm^2^)
A (16–20)	47.3 ± 8.2	48.0 ± 8.2	13,067 ± 1,799	15,433 ± 2,359
B (21–40)	47.4 ± 9.3	48.3 ± 9.4	13,647 ± 1,998	14,353 ± 2,423
C (41–60)	46.9 ± 9.4	48.1 ± 11.3	14,000 ± 2,194	14,212 ± 2,607
D (61–80)	46.7 ± 11.3	46.5 ± 13.6	12,296 ± 775	14,333 ± 2,922
*r*_s_ (*P*)	-0.084 (0.349)	-0.037 (0.683)	-0128 (0.154)	-0.156 (0.082)
*F* (*P*)	0.107 (0.956)	0.192 (0.901)	4.658 (0.004)	1.761 (0.158)

*ANOVA and Spearman correlation were used. SCn, nasal Schlemm’s canal; SCt, temporal Schlemm’s canal; CSAn, nasal cross-sectional area; CSAt, temporal cross-sectional area*.

## Discussion

In our study, we found that the positive detection rate of visible SC decreased with age in healthy subjects. The CSA was significantly larger on the temporal than on the nasal side. Though no significant correlation was observed between aging and SC parameters, we did find that the CSAn in Group D was significantly smaller compared with the other age groups.

With higher scanning resolution, SD-OCT has been proven to be a powerful tool for SC assessment when compared with other measuring instruments, such as ultrasound (McKee et al., 2013; Kagemann et al., 2014a,b). Forgáčová et al. (2013) investigated SCs of 92 eyes from 46 healthy volunteers in the horizontal meridian at 3 and 9 o’clock positions using time-domain OCT. They reported that the external and internal SC diameters were 381.75 and 380.65 μm, respectively. Wang et al. (2012) examined CSA and SC long diameter both nasally and temporally with swept source OCT in 74 normal participants, and the average measurements were 267.19 ± 51.56 μm and 278.30 ± 47.26 μm for nasal and temporal SC long diameters, 8,042.74 ± 1498.32 μm^2^ and 8005.56 ± 1,495.15 μm^2^ for nasal and temporal CSA, respectively. Researchers compared two SD-OCT devices (iVue and Cirrus) for the identification capability of angle structures and found equal frequency for both OCT devices when identifying SC microstructures (reviewed in Wang et al., 2012). In the current study, the average SC diameter and CSA values were 46.9 ± 9.6 μm and 13,296 ± 1897 μm^2^ in the nasal section and 47.8 ± 10.6 μm and 14,552 ± 2,589 μm^2^ in the temporal section. The relatively small diameter values in our study should be attributed to our protocol of measuring sagittal diameter of SC instead of the meridional or coronal diameter, as it is susceptible to confuse measurements of the meridional or coronal diameter with the collector channel (Hong et al., 2013). We found that the nasal CSA was significantly different in the four age groups.

Although the possibility of imaging the SC with SD-OCT has been reported, we still encountered some difficulties in detecting the SC because of its relatively small lumen size compared with other angle structures, such as the scleral spur (SS) or Schwalbe’s line (SL). Besides, shadows from superficial scleral blood vessels often obscure part or all of SC in the OCT imaging procedure, making the measurements more difficult. Kagemann et al. (2010, 2014b) applied the Biotogen SD-OCT to specifically image the SC and showed that all 21 normal individuals had detectable SC in the nasal and temporal areas. McKee et al. (2013) identified an average of 27% [range, 12% (inferior) to 42% (temporal)] of high-density scan images with visible SC. Nevertheless, most previous studies reported only 32–34% of eyes had visible SC (reviewed in McKee et al., 2013). In the current study, almost one fifth (30/155) of eyes enrolled initially were excluded from final data analysis because of unavoidable abnormalities of the eyes, such as pterygium (1/155), pinguecula (8/155), and dense arcus senilis (21/155) which affected clear visualization of SC. However, the positive rate of SC was still similar to our previous studies (Hong et al., 2013, 2016).

The mean CSA was found to be significantly larger in the temporal section than in the nasal section (14,552 vs. 13,296 μm^2^; *P* < 0.05). This is inconsistent with previous findings by Kagemann et al. (2014b; reviewed in Forgáčová et al., 2013) who noted that the mean CSA was significantly smaller in the temporal section in 21 healthy subjects (8,303 vs. 10,983 μm^2^; *P* = 0.009). Similarly, Li et al. (2017) reported greater CSA on the nasal side in 11 normal eyes (3,839 ± 1,402 mm^2^ vs. 3,189 ± 1,209 mm^2^; *P* = 0.033). Forgáčová et al. (2013) did not observe any significant differences in SC size between the nasal and temporal sides in their investigation of 62 POAG and 92 healthy eyes, either. This discrepancy among studies may be partly associated with the different imaging methods and racial differences.

Our study had several limitations. First, subjects younger than 16 years of age were excluded from the current study, so the variations of SC parameters in the entire age range were not available. Another limitation was that we only recruited Chinese participants. Thus, our findings may not be generalized to other regions.

## Conclusion

We found that the CSA was greater in the temporal section. Significant variations of nasal CSA were found among the four age groups. The positive detection rates of SC showed a significantly downward trend with age. Clinicians may need to take age-related changes of SC into account in therapeutic decision-making and outcome assessment of glaucoma surgeries when targeting SC.

## Author Contributions

XS, JX, and JH designed the study. JC and XY performed the experiments. YZ collected the data, performed the statistical analysis, and wrote this article.

## Conflict of Interest Statement

The authors declare that the research was conducted in the absence of any commercial or financial relationships that could be construed as a potential conflict of interest.
